# Electronic and optical properties of vacancy ordered double perovskites A_2_BX_6_ (A = Rb, Cs; B = Sn, Pd, Pt; and X = Cl, Br, I): a first principles study

**DOI:** 10.1038/s41598-021-86145-x

**Published:** 2021-03-26

**Authors:** Muhammad Faizan, K. C. Bhamu, Ghulam Murtaza, Xin He, Neeraj Kulhari, Murefah Mana AL‐Anazy, Shah Haidar Khan

**Affiliations:** 1 Department of Physics, University of Peshawar, Peshawar, 25120 Pakistan; 2 State Key Laboratory of Superhard Materials and School of Materials Science and Engineering, Jilin University, Changchun, 130012 China; 3 Physical and Materials Chemistry Division, CSIR-National Chemical Laboratory, Pune, 411008 India; 4 Department of Physics, Gramin Mahila P.G. College, Sikar, Rajasthan 332024 India; 5 Materials Modeling Lab, Department of Physics, Islamia College University, Peshawar, 25120 Pakistan; 6 Department of Mathematics & Natural sciences, Prince Mohammad Bin Fahd University, P. O. Box 1664, Alkhobar 31952, Saudi Arabia; 7 Department of Physics, IIS (Deemed To Be University), Jaipur, Rajasthan 302020 India; 8 Department of Chemistry, College of Science, Princess Nourah bint Abdulrahman University, Riyadh, 1167 Saudi Arabia

**Keywords:** Condensed-matter physics, Materials for devices, Materials for energy and catalysis, Photovoltaics

## Abstract

The highly successful PBE functional and the modified Becke–Johnson exchange potential were used to calculate the structural, electronic, and optical properties of the vacancy-ordered double perovskites A_2_BX_6_ (A = Rb, Cs; B = Sn, Pd, Pt; X = Cl, Br, and I) using the density functional theory, a first principles approach. The convex hull approach was used to check the thermodynamic stability of the compounds. The calculated parameters (lattice constants, band gap, and bond lengths) are in tune with the available experimental and theoretical results. The compounds, Rb_2_PdBr_6_ and Cs_2_PtI_6_, exhibit band gaps within the optimal range of 0.9–1.6 eV, required for the single-junction photovoltaic applications. The photovoltaic efficiency of the studied materials was assessed using the spectroscopic-limited-maximum-efficiency (SLME) metric as well as the optical properties. The ideal band gap, high dielectric constants, and optimum light absorption of these perovskites make them suitable for high performance single and multi-junction perovskite solar cells.

## Introduction

Initial studies on lead halide perovskite materials as light absorbers were published in 2009^1–3^. The power conversion efficiencies (PCEs) of these materials exceed 25% according to a recent report^4^. Such a fast improvement is attributed to the unique photovoltaic properties of Pb halide perovskite absorbers, for example, tunable direct band gap, fair electron and hole effective mass, excellent optical absorption, high stability, benign defect tolerance, and long term photogenerated carrier diffusion lengths^5–7^. The commercial use of perovskites containing Pb for photovoltaic applications has attracted a great deal of research interest^8^. However, two major challenges still exist: lead (Pb) is toxic, and the perovskites suffer from low chemical stability in air^9–12^. Different approaches have been used to combat the toxic/stability issue of the lead halide perovskites, such as using alternatives to lead^13,14^, fabricating 2D perovskites^15,16^ and using mixed cations^17–21^. Such modification in the structure will greatly affect the electronic structure and optical properties of perovskite compounds^22^. Taking into account both the crystal structure and chemical composition, novel materials for efficient photovoltaic application in the perovskite family could be discovered with the help of computational approach such as the density functional theory^23^.

The basic crystal structure of perovskite compounds is of the type ABX_3_ such that A is a monovalent and B a divalent cation, and X is an anion (e.g., CH_3_NH_3_PbI_3_ and CsSnI_3_)^17,24^. However, some modifications in the basic structure of perovskites, such as A_2_B^1+^B^3+^X_6_^17^ and A_2_B^4+^X_6_^17,25^, have been reported in literature. The A_2_B^1+^B^3+^X_6_ structure (also called elpasolite) can be considered as cubic by replacing every pair of adjacent B-cations with one B^1+^ and one B^3+^ cation at the top. Typical examples of this kind of the compounds are Cs_2_BiAgBr_6_^26^, Cs_2_SbAgCl_6_^27^, and Cs_2_AgInCl_6_^28^. Structure-wise, the A_2_BX_6_ perovskites such as Cs_2_SnI_6_ or Cs_2_PdBr_6_, can be considered as a derivation from ABX_3_ if half of the B cations at the [BX_6_] cluster center are removed in a checkerboard type pattern^17^. The charge neutrality condition implies that the B-site cation should be a tetravalent, extending the type of cations for substitution into B-site^29^. Generally, it is referred to as antifluorite crystal (K_2_PtCl_6_) and is described by the [$BX_{6}$]^2–^ octahedral cluster bridged by the A-site cations^25^. The A_2_BX_6_ structure shows similar features to ABX_3_ perovskites and most of them possesses cubic structure.

Recently, the Cs_2_BI_6_ with B = Sn and Te have been reported capable of absorption of light in the visible to infrared (IR) region giving new hope for stable materials with a nature friendly operation^25,30,31^. In this framework, Cs_2_SnI_6_ with cubic crystal structure containing Sn in its +4 oxidation state is regarded as a potential candidate for applications in perovskite solar cells (PSCs)^30^. Diffuse-reflectance measurements of Cs_2_SnI_6_ show an optical band gap of 1.25–1.30 eV in comparison with the thin films band gap of 1.60 eV. The density functional theory results in a direct transition nature with the suggested band gap (0.13 to 1.26 eV) significantly different from the experimental value given above, most likely using functionals with different correlation approximations^29,32^. The compound possesses N-type electrical conductivity, strong absorption power, and moisture stability^30,31,33^. Due to the tetravalent character of Sn, Cs_2_SnI_6_ exhibits higher air stability with respect to CsSnI_3_^29^. In fact, numerous non- or low-toxic transition metals have stable +4 oxidation state paving the way for finding favorable halide perovskites, for example by replacing the Sn^4+^ in Cs_2_SnI_6_ by appropriate transition-metal cations^34^. This is confirmed by Sakai et al. who studied Cs_2_PdBr_6_ as a novel perovskite for application in PSCs^35^. The optical band gap of Cs_2_PdBr_6_ calculated from absorption measurement was 1.60 eV^35^. The effective mass of electrons and holes calculated from first principles technique were reported to be 0.53 m_e_ and 0.85 m_e_, which indicate an N-type semiconducting behavior for Cs_2_PdBr_6_^35^. Ju et al. carried out an integrated experimental and theoretical study of Ti-based vacancy-ordered double perovskites (DPs) A_2_TiX_6_ (A = K^+^, Rb^+^, Cs^+^ In^+^; X = I, Br, or Cl) and Cs_2_TiI_x_Br_6−x_, showing a suitable band gap in the range from 1.38 to 1.78 eV for photovoltaic applications^34^. Zhao et al. studied a new family of vacancy ordered DPs, Cs_2_BX_6_ (B = Pd, Sn, Ti, Te; X = Cl, I), claiming the compounds possess diverse electronic structures and optical features^36^. A group of researchers computationally studied compounds of the type A_2_MX_6_ (A = K, Rb, and Cs, M = Sn, Pd, Pt, Te, and X = I) by using hybrid functional (HSE06), reporting the variation of band gap and effective mass with the A-site cation changing from K to Rb to Cs^8^.

To conduct further research aimed at the use of various metals substitution for photovoltaic and optoelectronic applications, we make use of the density functional theory to explore new variants in A_2_BX_6_ family with possible A = Rb, Cs; B = Sn, Pd, or Pt; and X = Cl, Br, or I. We begin from the structural properties and then examine the electronic structure as well as optical spectra of these compounds. In addition, we also report on the thermodynamic stability of these compounds by calculating their formation energies.

## Computational methods

Structures and other physical properties of existing as well as hypothetical compounds can be approximated with considerable success using the density functional theory. To manipulate the vacancy-ordered DPs, we used wien2k^37^ code based on the density-functional theory (DFT)^38^ by employing the Full Potential Linearized Augmented Plane Wave (FP-LAPW) method with Perdew, Burke, and Ernzerhof functional (PBE)^39^, modified Becke Johnson (mBJ) semi-local exchange potential^40^, and hybrid functional HSE06^41^. The band structure and the density of states were further examined taking into consideration the spin–orbit coupling (SOC)^42^ interaction. In HSE06 calculations, we used 25% of the exact Hartree–Fock exchange fraction together with 75% exchange and 100% correlation energies of PBE functional. Both the mBJ and HSE06 have been shown to produce more accurate band gap as compared to standard LDA/GGA functionals^43,44^. Further details about the computational technique can be found in the supplementary information.

## Results and discussion

### Structural properties

**Figure 1:**
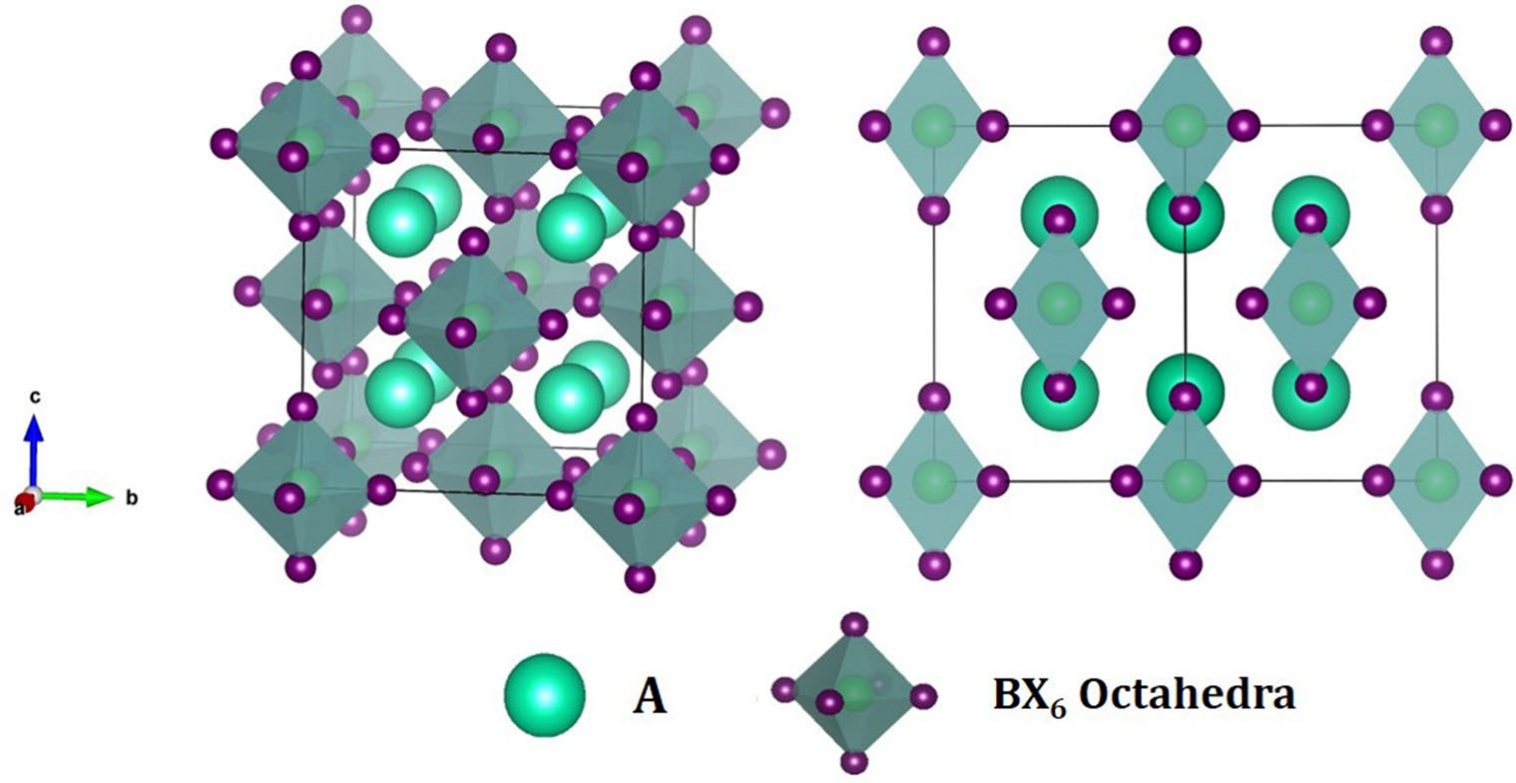
Schematic crystal structures of A_2_BX_6_ compounds in cubic ${\text{Fm}}\overline{3}{\text{m}}$ (Left) and reorientation of the unit cell (right). The BX_6_ octahedra are shaded, with the X-atom on the corners. The A-cations are in the hole between BX_6_ octahedra.

Our calculations show that the vacancy-ordered DPs A_2_BX_6_ (A = Rb and Cs; B = Sn, Pd, and Pt; and X = Cl, Br, and I) have a face centered cubic structure with space group ${\text{F}}m\overline{3}m$ (No. 225). The atomic positions and geometric configuration of A_2_BX_6_ is illustrated in Fig. 1 which can be described as B-deficient ABX_3_ perovskites with [BX_6_] cluster. The vacant sites between [BX_6_] octahedra are filled with A-site atoms. Each [BX_6_] octahedra in A_2_BX_6_ structure is isolated from the others forming a 12-fold coordination environment of discrete X anions. The A-cations are surrounded by twelve and the B atoms by six halogens ions. The [BX_6_] octahedra form a cubic environment, placed at the corners and at the face center positions. The A-site atoms are located at 8c Wyckoff site and (1/4, 1/4, 1/4) fractional coordinates, the B-site cations are located at 4a Wyckoff site and (0, 0, 0) coordinates, and the X-anions lie at 24e Wyckoff site and (x, 0, 0) fractional coordinates. The variable x lies close to 0.2. The energy-volume optimization curve was calculated by fitting the Murnaghan’s equation of state (EOS) to the PBE-GGA total energy (Fig. S1, in Supplementary Information). The calculated lattice parameters as compared to experimental results are listed in Table 1. The lattice constants are found in good agreement with experiments showing an increasing trend with changing Cl to Br and then to I in agreement with their changing geometry (Fig. S2, in Supplementary Information). The calculated bond lengths obtained after energy minimization for A_2_BX_6_ compounds are presented in Supplementary Information Table S1. The phase stability was also assessed using the tolerance factor ‘*t*’ proposed by Goldschmidt^45^ and was found well within the proposed range (0.8 ≤ *t* ≤ 1.11)^46^ for stable 3D cubic halide perovskites, as shown in Table 1.

**Table 1:** Optimized lattice constant and unit cell volume of A_2_BX_6_ (A = Rb, Cs; B = Sn, Pd, Pt; X = Cl, Br, I) using the PBE functional.

Compound	a (Å)	Exp. (Å)	Volume/Å^3^	*t*_eff_
Rb_2_SnBr_6_	11.01	10.58^47^	1193.98	0.98
Rb_2_SnI_6_	11.87	11.62^48^	1493.78	0.96
Rb_2_PdCl_6_	10.23	9.990^49^	956.84	1.02
Rb_2_PdBr_6_	10.71	10.02^50^	1098.80	1.01
Rb_2_PdI_6_	11.48	11.185^51^	1351.18	0.99
Cs_2_PtCl_6_	10.62	10.192^52^	1070.03	1.07
Cs_2_PtBr_6_	11.06	10.670^53^	1209.78	1.05
Cs_2_PtI_6_	11.77	11.367^54^	1457.65	1.02

**Figure 2:**
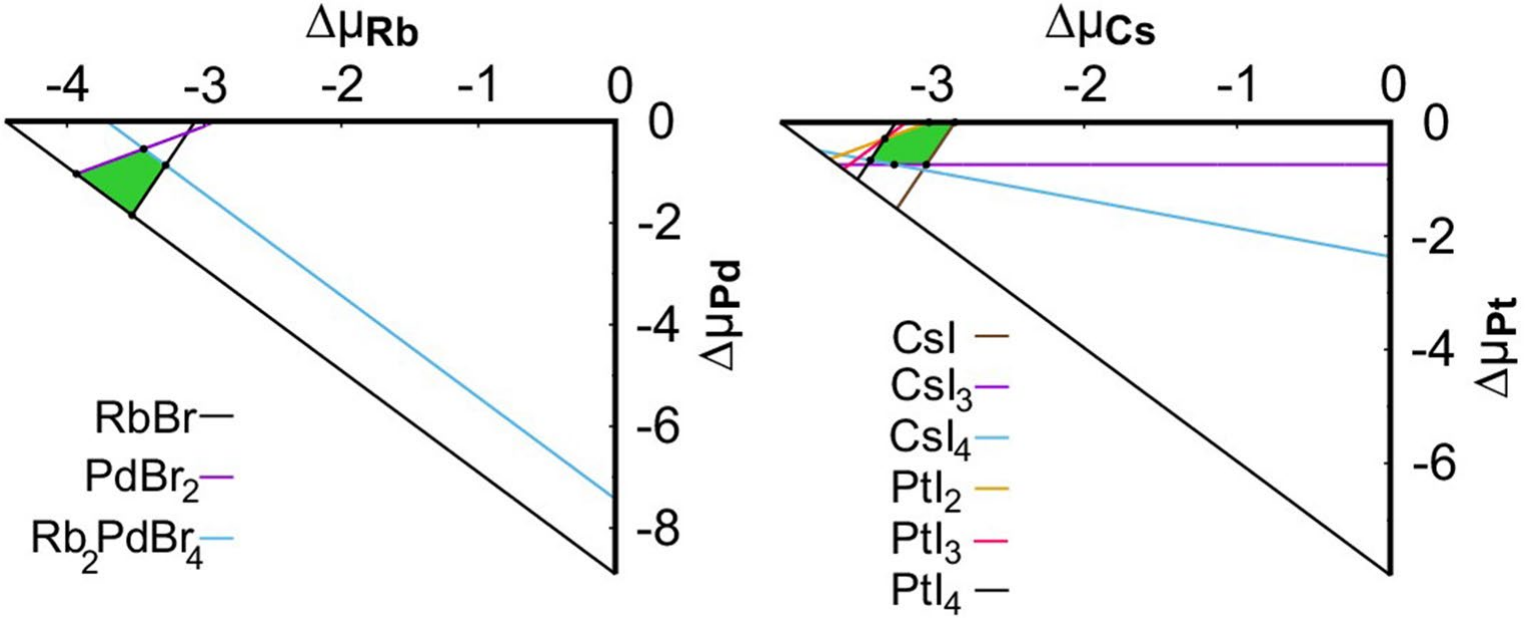
The stability diagrams of Rb_2_PdBr_6_ (left) and Cs_2_PtI_6_ (right). Each line in the diagrams indicates a known competing phase; in each case the stable region is indicated by the green polygon.

The thermodynamic stability of the compounds was explored via the commonly used convex hull approach (details are given in the Supplementary Information). The full list of the competing phases as well as the calculated formation energies for the set of the compounds are given in Table S2. The accessible range of their chemical potentials in a two dimensional plane is shown in Figs. 2 and S3 (Supplementary Information). For each compound, all the relevant binary and ternary phases were considered. Among the eight compounds, only five were found as potentially stable.

### Band structure and density of states

**Figure 3:**
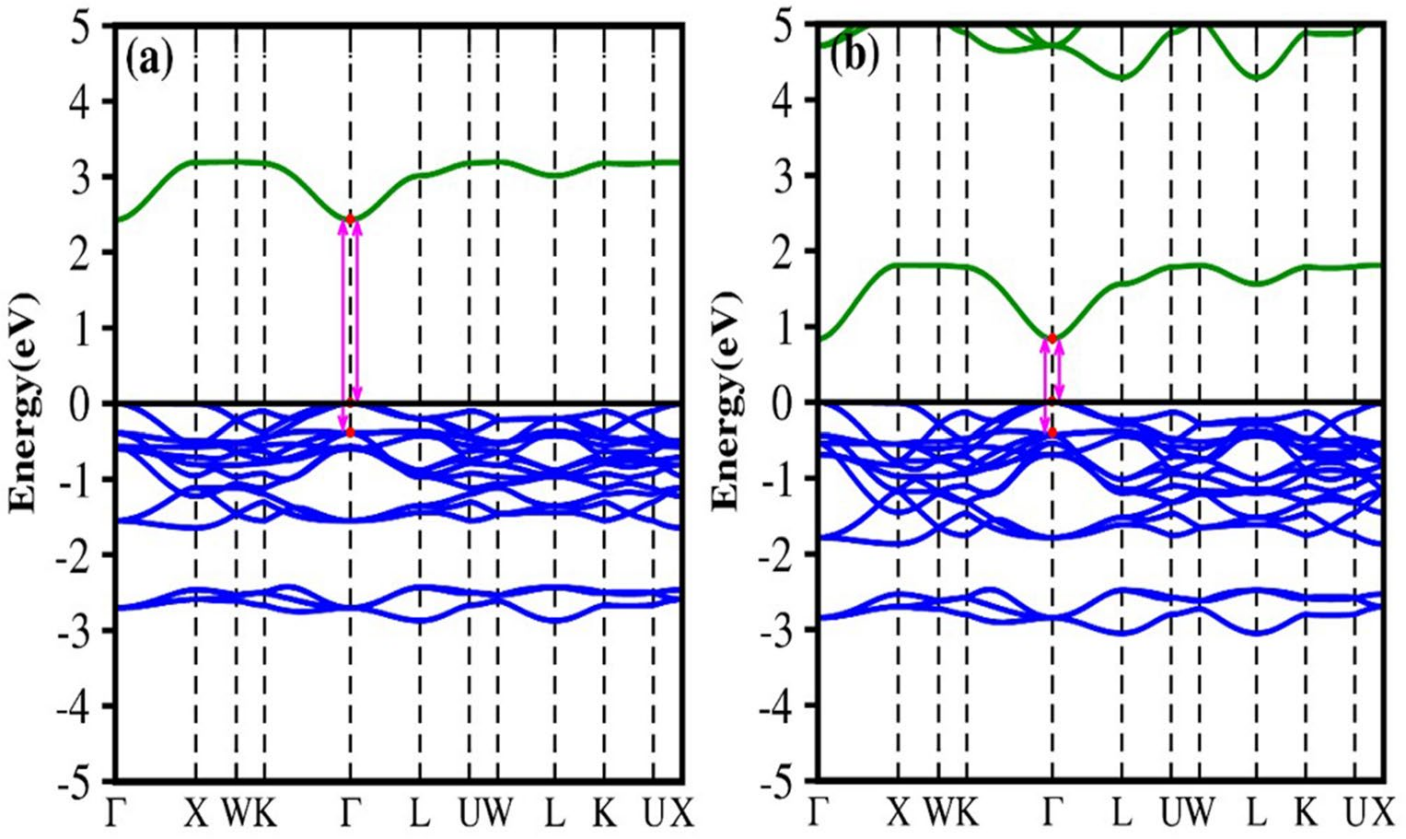
The calculated band structure of (**a**) Rb_2_SnBr_6_ and (**b**) Rb_2_SnI_6_ with mBJ potential.

**Figure 4:**
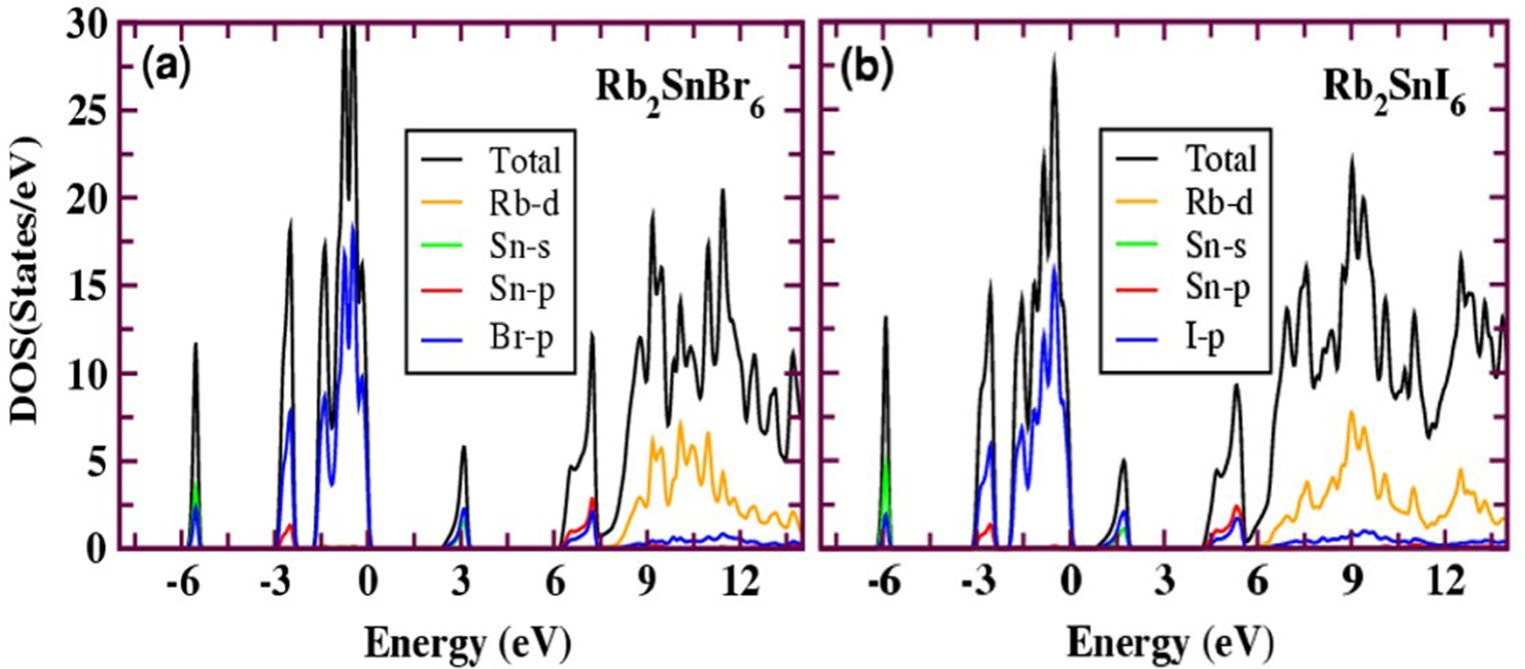
The total and partial density of states (DOS) for (**a**) Rb_2_SnBr_6_ and (**b**) Rb_2_SnI_6_ computed using mBJ potential. The Fermi level is set to 0 eV.

Generally, the band gap of inorganic Pb-free perovskites solar material ought to be within the optimal range: 0.9 to 1.6 eV (efficiency > 25%)^55^. The band gap calculated with various exchange–correlation functionals is given in Table 2. The mBJ calculated band gap ranges from 0.47 to 2.91 eV with the lowest for Rb_2_PdI_6_ and the highest for Cs_2_PtCl_6_. The calculated mBJ band structures for A_2_BX_6_ are shown in Figs. 3, S4 and S5 (Supplementary Information). Each figure gives the band structure for a fixed A-site cation along the high symmetry direction in the Brillouin zone, suggesting a semiconducting characteristic. Both Rb_2_SnBr_6_ and Rb_2_SnI_6_ show a direct band gap nature (Fig. 3). The valence band maximum (VBM) and the conduction band minimum (CBM) are localized at the Г (0.0, 0.0, 0.0) symmetry point. The band gap calculated using mBJ are 2.42 eV (Rb_2_SnBr_6_) and 0.84 eV (Rb_2_SnI_6_) slightly larger than that calculated with PBE-GGA. When we include the spin–orbit coupling (SOC) effect, the computed band gap for Rb_2_SnI_6_ reduces by 0.16 eV bringing it to 0.68 eV. The mBJ calculated band gap seems to be significantly underestimated as compared with a UV–visible experiment (1.32 eV)^56^ and a calculated (HSE06) value for cubic (1.02 eV)^8^ and tetragonal (1.13 eV)^56^ phase. However, our calculated HSE06 values are close to the experiment as well as other theoretical works (Table 2). For these compounds, the valence band is mainly derived from Sn-5*s* and X-5*p* anti-bonding orbitals whereas the conduction band is derived entirely from Sn-5*p* and Rb-3*d* anti-bonding orbitals as shown in Fig. 4. For the Pd-based compounds, Rb_2_PdCl_6_, Rb_2_PdBr_6_, and Rb_2_PdI_6_, the computed band structures are shown in Fig. S4. The plots reveal indirect band gap character for all the three compounds between the VBM and CBM at Г (0.0, 0.0, 0.0) and X (0.0, 0.5, 0.5) symmetry lines. We see from the figure that with fixed A and B site cations, the band gap decreases by replacing Cl with Br and I. This trend may be due to the decrease of electronegativity difference between B-site elements (Pd@2.2) and halide ions (Cl@3.16, Br@2.96, and I@2.66) which increases the Pd-X covalent strength and makes the valence band more dispersive (shown in Fig. S4). This, in turn, can push the Pd-X anti-bonding orbitals making the VBM higher in energy resulting in a smaller band gap. The effect of SOC is insignificant in the Pd-based compounds. The PBE-GGA method predicts the Rb_2_PdI_6_ compound as metal, however, both the mBJ and mBJ-SOC predicts it a narrow band gap semiconductor. The chosen k-path for this series of the compounds is *Г-X-W-K-Г-L-U-W-L-K-U-X*. The bands are nearly parabolic in the Г-K and Г-L directions which is helpful to enhance the carrier mobility while the bands in Г-X direction are relatively flat. The calculated density of states (DOS) shown in Fig. S6 depicts that the valence band is primarily formed due to the mix contribution of Pd-*d* and Cl/Br/I-*p* orbitals while the conduction band is formed due to the Cl/Br/I-*p* and Rb-*d* orbitals. Some bonding interaction can be seen in the lower part of the VBM between Pd-*d* and Cl/Br/I-*p* states. Strikingly, in Pd-based compounds there are triply degenerate states at VBM along the Г-X directions. Such states around the VBM have a profound effect on the thermoelectric properties.

**Table 2:** Band gap calculated by different GGA exchange-correlation functionals for A_2_BX_6_ (A = Rb, Cs; B = Sn, Pd, Pt; X = Cl, Br, I) compounds.

Compound	PBE	mBJ	mBJ-SOC	HSE06	Other works	Exp:
Rb_2_SnBr_6_	1.30	2.42	2.42	2.17		
Rb_2_SnI_6_	0.12	0.84	0.68	1.10	1.02^8^	1.32^56^
Rb_2_PdCl_6_	1.28	2.20	2.20	2.25		
Rb_2_PdBr_6_	0.61	1.31	1.31	1.39		
Rb_2_PdI_6_	0.0	0.47	0.39	0.56		
Cs_2_PtCl_6_	2.01	2.91	2.71	3.30		
Cs_2_PtBr_6_	1.42	2.23	2.21	2.31		
Cs_2_PtI_6_	0.65	1.22	1.11	1.32	1.34^8^

Figure S5 shows the band structure of Cs_2_PtCl_6_, Cs_2_PtBr_6_, and Cs_2_PtI_6_. The band gap seems to increase in the order Cs_2_PtCl_6_ > Cs_2_PtBr_6_ > Cs_2_PtI_6_, consistent with the trend observed in CH_3_NH_3_PbX_3_ (X = Cl, Br, I)^57^. Only the Cs_2_PtCl_6_ perovskite possesses a direct band gap (Fig. S5) at the X symmetry point. The calculated direct gap ($E_{g}^{{{\text{X}} - {\text{X}}}}$) is 2.91 eV (mBJ) and 2.71 eV (mBJ-SOC), respectively. The Cs_2_PtBr_6_ reveals an indirect band gap of 2.23 (mBJ) and 2.21 eV (mBJ-SOC) showing a poor spin–orbit coupling effect. 

For Cs_2_PtI_6_, our calculations show an indirect band gap of 1.22 and 1.11 eV (mBJ and mBJ-SOC) at *Г − X* symmetry lines. The mBJ based band gap is in a better agreement with an earlier report $(E_{g}$ = 1.34 eV)^8^ determined using HSE-SOC functional, justifying using the mBJ approximation in our calculations. The lower part of the valence band is predominantly formed by Cs-*p* (~ –7 eV) whereas the VBM is mainly due to Cl/Br/I-*p* states (Fig. S6). The conduction bands are composed of the Pt-*d* and halogens-*p* states. Interestingly, we found that among the eight semiconductors, Rb_2_PdBr_6_ (1.31 eV) and Cs_2_PtI_6_ (1.22 eV) have favorable band gap in the optimal range of 0.9–1.6 eV, suggesting their possible use in single-junctions PSCs. These findings are also supported by our calculated favorable effective mass for electrons and holes for Rb_2_PdBr_6_ and Cs_2_PtI_6_ as shown in Table S3. We used single parabolic band approximation to calculate the carrier effective mass for these compounds. Despite the favorable band gap and the effective mass, the Pt and Pd based compounds show multiple minima (at X and K/U symmetry points) in the conduction band. The energy difference between the two minima is ~ 0.05 eV which gives rise to the valley degeneracy responsible for the high figure of merit ^58^. This difference can be eliminated by strain engineering. It is reported in literature^58–60^ that the band convergence improves the thermoelectric performance (TEP) of the system, thus we can also predict that with applying the suitable strain in Pt and Pd based compound, their TEP can be improved. However, calculating TEP of the present class of materials is beyond the scope of the present investigation. Hopefully, our observation may motivate the scientific community to consider working on TEP of Pt and Pd based compounds.

### Optical properties

**Figure 5:**
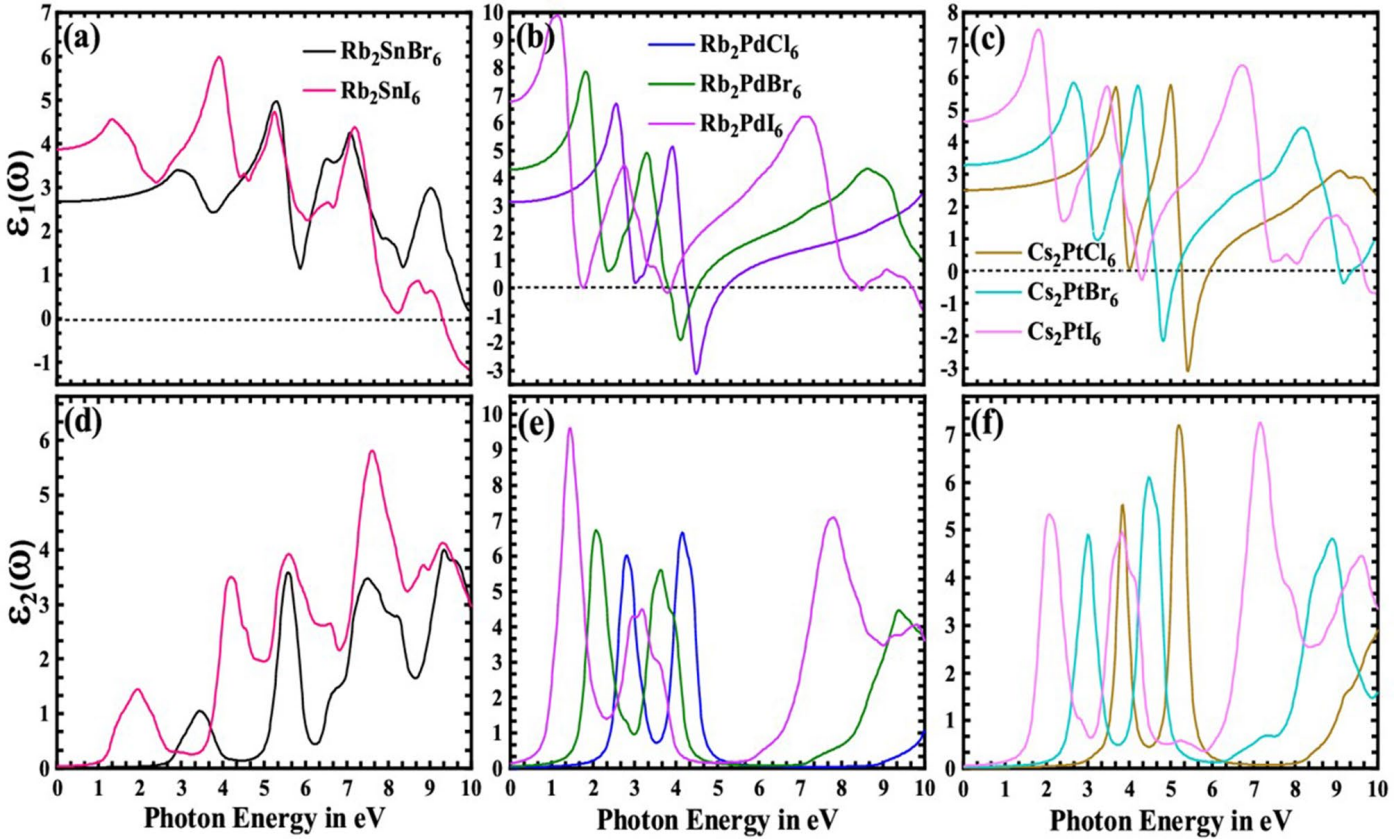
Real (**a**–**c**) and imaginary (**d**–**f**) part of the dielectric function for A_2_BX_6_ (A = Rb, Cs; B = Sn, Pd, Pt; X = Cl, Br, I) computed using mBJ potential.

The computed real $\varepsilon_{1} \left( \omega \right)$ and imaginary $\varepsilon_{2} \left( \omega \right)$ part of the dielectric function are depicted in Fig. 5 and Supplementary Table S4. The static dielectric constant $\varepsilon_{0}$ of Rb_2_PdI_6_ was found to be 6.76, which is larger than that of the Pb-based CH_3_NH_3_PbI_3_ perovskite (~ 5.2)^61^. A large value of the static dielectric constant is essential for efficient light absorption. It can promote low level of charge defects and prohibit radiative electron–hole recombination rate. Further, the real part, $\varepsilon_{1} \left( \omega \right)$, increases to a maximum (9.9 for Rb_2_PdI_6_ at 1.15 eV) and then decreases asymptotically to negative values making re-inversions to secondary maxima and minima. From Fig. 5a–c, it is obvious that the curves are redshifted towards the visible region by changing the halide ions (Cl → Br → I) across the selected compounds, causing an increase in $\varepsilon_{1} \left( \omega \right)$ and shifting of peaks towards the low energies.

The imaginary parts $\varepsilon_{2} \left( \omega \right)$ of the dielectric function in Fig. 5d–f exhibits the optical transitions between VBM and CBM at the threshold energy 2.41 (Rb_2_SnBr_6_) and 0.86 eV (Rb_2_SnI_6_), which are direct as evident from the band structure plot (Fig. 3). For Rb_2_PdCl_6_, Rb_2_PdBr_6_, and Rb_2_PdI_6_, the threshold is 2.12, 1.33, and 0.49 eV, respectively, with indirect transition between the VBM and the CBM. Similarly, the thresholds for Cs_2_PtCl_6_, Cs_2_PtBr_6_, and Cs_2_PtI_6_ are 2.93, 2.08 and 1.11 eV, in agreement with the calculated band gap (Table 2) having indirect transition. Above the threshold points, there are different peaks with some noticeable variations. The maximum peak value of $\varepsilon_{2} \left( \omega \right)$ for all the compounds are given in Table S4. It is obvious from Fig. 5d–f and Table S4 that the computed intensities of $\varepsilon_{2} \left( \omega \right)$ for the iodide-based compounds are higher than those of the Br- and Cl-containing compounds. The main reason is that the band gap of the former phases is smaller than the latter structures. Hence, according to the Fermi golden rule, the transition probability is reduced in the Br- and Cl-based compounds.

**Figure 6:**
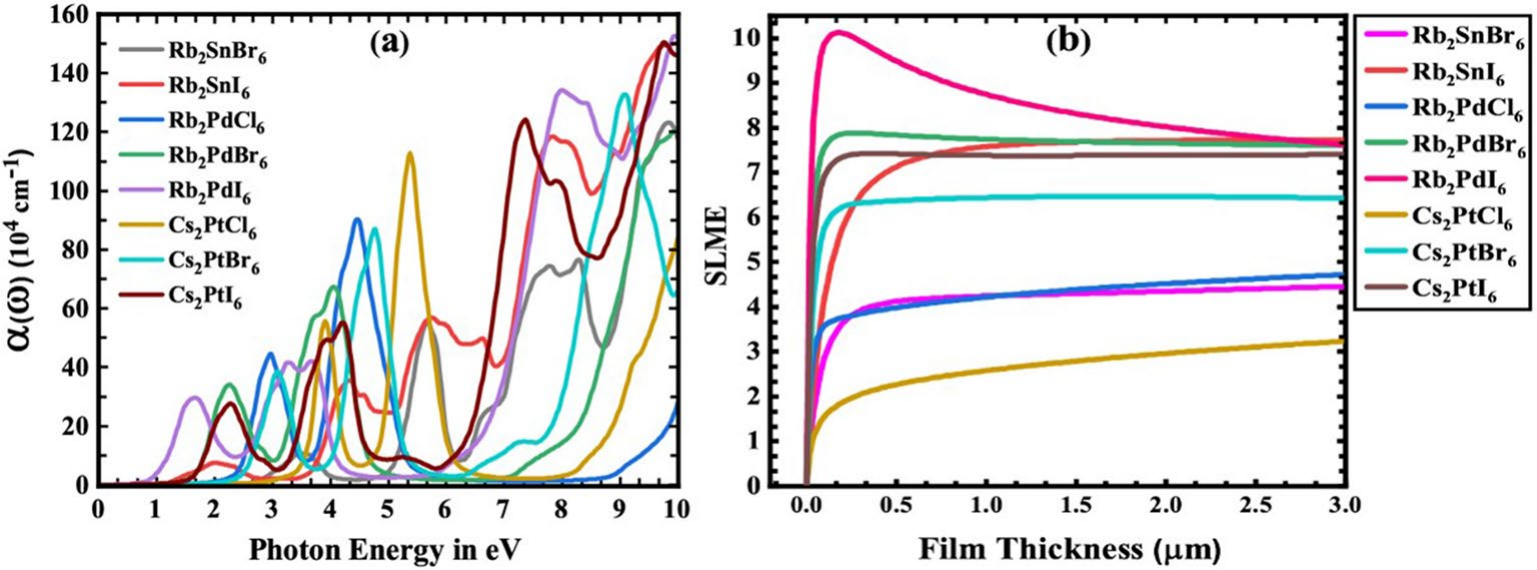
(**a**) Calculated absorption coefficient and (**b**) SLME for A_2_BX_6_ (A = Rb, Cs; B = Sn, Pd, Pt; X = Cl, Br, I) compounds.

Figure 6a shows the calculated absorption coefficients $\alpha \left( \omega \right)$ with the absorption edges at 0.5–2.9 eV for different compounds in reasonable agreement with the corresponding band gap (mBJ). Several absorption peaks with increasing trend can be attributed to the electronic transitions from bonding states to the anti-bonding states. In the visible energy range, the maximum absorption (5.71 × 10^5^ cm^−1^) corresponds to Rb_2_PdBr_6_ perovskite. Therefore, Pd can be considered a suitable alternative to Pb in inorganic perovskites solar cells. Within this group (Rb_2_PdX_6_), the maxima of the absorption peaks have a significant downward trend in unison with photon energy as a function of increasing halogen radii. The rest of the compounds have peaks in the ultraviolet region suitable for optical devices working in this range. The optical conductivity as well as reflectivity spectra have also been calculated, their full details is provided in the Supplementary Information (Figs. S7, S8).

A successful criteria for assessing the photovoltaic efficiency of a solar absorber is the spectroscopic-limited-maximum-efficiency (SLME)^62^. It takes into account the band gap, the optical absorption spectrum, the recombination mechanism, and the fundamental transition. The computed SLME for our compounds are shown in Fig. 6b. The SLME of Rb_2_PdBr_6_, Rb_2_PdI_6_, and Cs_2_PtI_6_ are higher than the rest of the compounds which can be attributed to their favorable band gap and optimum light absorption.

## Conclusions

We have performed first-principles calculations employing the mBJ potential to investigate the electronic structure as well as the optical properties of defect perovskites A_2_BX_6_ (A = Rb and Cs; B = Sn, Pd, and Pt; and X = Cl, Br, and I). The structural analysis shows a monotonic increase of the lattice constant and volume by changing the halide ion from Cl to Br and then to I which results in a gradual increase in the B-X bond length. The calculated enthalpies of formation for the investigated A_2_BX_6_ family are found to be negative, except for Rb_2_SnBr_6_, Rb_2_SnI_6_, and Rb_2_PdI_6_, and also the calculated tolerance factor of all the compounds ranges from 0.96 to 1.07, lying within the specified range for stability of the cubic halide perovskites. The results show that all the compounds possess optimum electronic and optical properties to be used as visible-light absorbing materials for PV applications. We also applied different exchange–correlation functionals, namely PBE-GGA, mBJ, mBJ-SOC, and HSE06 to get a close approximation of the true band gap of the A_2_BX_6_ family. The HSE06 functional gives the nearest approximation to the available experimental results. The band gap varies most likely due to the electronegativity or size difference of B- and X-site atoms. Among the entire group of the compounds studied, the ideal band gap was obtained only for Rb_2_PdBr_6_ (1.31 eV) and Cs_2_PtI_6_ (1.22 eV) in the optimal range of 0.9–1.6 eV. This indicates that both the compounds are potential candidates for single-junction solar cell application in the future. We also calculated different optical properties, more specifically, the complex dielectric function, absorption coefficient, and SLME which support the use of these materials in various optoelectronic applications. The compounds, Rb_2_PdBr_6_, Rb_2_PdI_6_, and Cs_2_PtI_6_, possess suitable band gap and relatively high optical absorption as compared to other members of the A_2_BX_6_ family. We hope that our results will motivate further research in this direction in order to use lead-free perovskite variants in efficient photovoltaic or other optoelectronic devices.

## Supplementary Information

Supplementary Information.
