# Supplementary material for: Electronic and optical properties of vacancy ordered double perovskites A2BX6 (A = Rb, Cs; B = Sn, Pd, Pt; and X = Cl, Br, I): a first principles study
Source: Sci Rep. 2021 Mar 26;11:6965. doi: 10.1038/s41598-021-86145-x (PMC7997874; doi:10.1038/s41598-021-86145-x)
Supplement: Supplementary file 1 — Supplementary Information. [file 41598_2021_86145_MOESM1_ESM.pdf]

Supplementary Information For:

Electronic and Optical Properties of Vacancy Ordered Double Perovskites A_2BX_6 (A = Rb, Cs; B = Sn, Pd, Pt; and X = Cl, Br, I): A first principles study

Muhammad Faizan[1,2*], K. C. Bhamu[3,4], Ghulam Murtaza[5,6], Xin He[2], Neeraj Kulhari[7], Murefah mana AL-Anazy[8], Shah Haidar Khan[1*]

[1]Department of Physics, University of Peshawar, Peshawar 25120, Pakistan

[2]State Key Laboratory of Superhard Materials and School of Materials Science and Engineering, Jilin University, Changchun 130012, China

[3]Physical and Materials Chemistry Division, CSIR-National Chemical Laboratory, Pune 411008, India

[4]Department of Physics, Gramin Mahila P.G. College, Sikar 332024, Rajasthan, India

[5]Materials Modeling Lab, Department of Physics, Islamia College University, Peshawar 25120, Pakistan

[6]Department of Mathematics & Natural sciences, Prince Mohammad Bin Fahd University, P. O. Box 1664, Alkhobar 31952, Kingdom of Saudi Arabia

[7]Department of Physics, IIS (Deemed to be University), Jaipur 302020, Rajasthan, India

[8]Department of Chemistry, College of Science, Princess Nourah bint Abdulrahman University, 1167, Riyadh, Saudi Arabia

*Correspondence: faizanstd@uop.edu.pk, shkhan@uop.edu.pk

1. Computational Detail

In this work, DFT-based first principles calculations were performed for perovskite derivative compounds (A_2BX_6) using wien2k simulation code. The relaxation of the size, shape and the relative atomic positions of the unit cells was done with an energy cutoff of 400 Ryd for plane wave expansion and ended when the energy become within 10^{-4} Ryd. For self-consistent calculations, the convergence criteria for the charge and atomic force were set at 0.001 e and 0.05 mRy/a.u, respectively. The convergence with respect to basis size is through the parameter, $R_{MT}K_{max}$, which is the product of the smallest muffin-tin sphere radius (R_{MT}) times the largest plane wave vector (K_{max}). The electronic and optical properties computations were performed with 5000 k-points. The other parameters, $G_{max} = 12\ a.u^{-1}$ (plane wave cutoff) and muffin-tin radii for Rb, Cs, Sn, Pd, Pt, Cl, Br, and I atoms were selected as 2.5 a.u for Rb, 2.5 a.u for Cs, 2.07 a.u for Sn, 2.11 a.u for Pd, 2.21 a.u for Pt, and 1.71/1.83/1.91 a.u for Cl/Br/I respectively. For all the compounds, the lattice constants and atomic positions from experimental data were used to perform the calculations. Calculations with finer k-points and high energy cutoffs confirmed the convergence of the results on the lattice parameters and electronic band structure.

2. Calculated bond lengths and tolerance factor

The bond lengths obtained after geometry optimization for A_2BX_6 (A= Rb, Cs; B= Sn, Pd, Pt; X= Cl, Br, I) compounds are listed in Table S1 along with the available experimental results. Due to the increase in the ionic radius of the halogens, the bond lengths A-X and B-X show an increasing trend similar to the lattice constant. Among the eight compounds, the Rb_2SnI_6, Rb_2PdI_6, and Cs_2PtI_6 possess larger bond lengths and thus has a larger lattice parameter. In fact, large bond lengths provide better explanation for their small band gap among the investigated A_2BX_6 perovskite.

The formation and distortion of perovskites structure is usually determined by the tolerance factor (t), introduced by V. M. Goldschmidt[1]. It can be estimated using the following expression:

$$t = \frac{(r_A + r_X)}{\sqrt{2}(r_B + r_X)}$$

where r_A is the radius of A-site cation r_B is the radius of B-site cation and r_X represent the ionic radius of X-site anion in A_2BX_6 structure. We have considered the Shannon effective ionic radii

for 12-coordinated A^+ cations, 6-coordinated B^{4+} cations, and 6-coordinated X^{1-} anions for r_A, r_B, and r_X, respectively[2]. Generally, the range of $0.8 \leq t \leq 1.11$[3,4] is usually considered a very good fit for halide perovskites and favors stable 3D cubic structure. According to the empirical conditions, when t is between 0.97 and 1.03[5], a crystal structure can reach an ideal perovskite model. For $t <$ 0.8, orthorhombic or rhombohedral structures most likely to form with tilted and distorted BX_6 octahedra while for $t > 1$, hexagonal or some non-perovskite structure can form [1,6]. Our calculated tolerance factor values are listed in Table 1; the values are in the range where the perovskite phase is stable and gives indication that these perovskites can be formed. Note that, the tolerance factors of Rb_2SnBr_6, Rb_2PdBr_6, Rb_2PdI_6, and Cs_2PtI_6 compounds are close to that of the empirically ideal perovskite structure.

3. Thermodynamic Stability

In order to assess the thermodynamic stability of each compound, their experimentally known competing phases were considered and the total energies were calculated for each phase. Further, the thermodynamic stability analysis require a stable A_2BX_6 compound (say Rb_2PdBr_6) to satisfy the following necessary criteria:

$$2\Delta\mu_{Rb} + \Delta\mu_{Pd} + 6\Delta\mu_{Br} = \Delta H(Rb_2PdBr_6) \qquad (1)$$

$$\Delta\mu_i \leq 0, \ (i = \text{Rb, Pd, Br}) \qquad (2)$$

$$n_j\Delta\mu_{Rb} + m_j\Delta\mu_{Pd} + q_j\Delta\mu_{Br} \leq \Delta H_f\left(Rb_{n_j}Pd_{m_j}Br_{q_j}\right), j = 1, \dots, Z \quad (3)$$

Where ΔH_f is the enthalpy of formation and $Rb_{n_j}Pd_{m_j}Br_{q_j}$ represents the total number of competing phases. Equ. (1) is for the equilibrium growth of a compound, equ. (2) is used to avoid precipitation into solid elemental Rb, Sn, and Br, and equ. (3) further constrained the chemical potential in order to avoid decomposition into possible competing phases spontaneously. Eqn.3 also ensures the energetic stability of Rb_2PdBr_6 against a wide range of binary and ternary phases. Table S2 list the formation energy of the relevant binary and ternary phases for the investigated stable compounds.

4. Optical Properties

To examine the performance of the A_2BX_6 (A= Rb and Cs; B = Sn, Pd, and Pt; and X= Cl, Br, and

I) perovskite when exposed to light, we have performed the first principles calculations for the dielectric and optical properties. The properties like complex dielectric function, optical absorption coefficient, optical conductivity, and reflectivity are investigated up to the photon energy of 10 eV to reveal the use of these materials in optoelectronic energy devices.

The optical conductance $\sigma(\omega)$ of Rb_2SnX_6(X= Br, I) starts around 0.77 and 2.34 eV, of Rb_2PdX_6 (X= Cl, Br, I) around 0.56, 1.13, and 1.97 eV (Fig. S7). Similarly, for Cs_2PtX_6 (X= Cl, Br, I), the optical conductivity activates at 2.7, 2.3, and 1.5 eV, respectively. The maximum optical conductivity is 7443 Ω^{-1} cm^{-1} and 6985 Ω^{-1} cm^{-1}corresponding to Rb_2PdI_6 and Cs_2PtI_6, respectively. For other compounds, the maximum conductivity values are recorded in Table S4.

The reflectivity spectra 'R(ω)' is given in Fig. S8 and Table S4. The static reflectivity lies in the range 5-19 % while the dynamic reflectivity increases with photon energy, peaking at ~5 eV. Rb_2PdCl_6 and Cs_2PtCl_6 show the two highest maxima: 64% (4.7 eV) and 54% (5.6 eV) close to $CH_3NH_3PbBr_3$[7]. The maximum reflectivity occurs in agreement with negative $\varepsilon_1(\omega)$ suggesting the use of these compounds as shield from ultraviolet radiations.

Table S1. Calculated bong lengths for A_2BX_6 (A= Rb, Cs; B= Sn, Pd, Pt; X= Cl, Br, I) compounds.

Compound	A-B(Å)		A-X (Å)		B-X (Å)	
	optimized	Exp.	optimized	Exp.	optimized	Exp.
Rb_2SnBr_6	4.7707		3.8966		2.6524	
Rb_2SnI_6	5.1406		4.1978		2.9061	2.85[8]
Rb_2PdCl_6	4.4297		3.6227		2.3513	
Rb_2PdBr_6	4.6405		3.7928		2.5079	
Rb_2PdI_6	4.9716		4.0620	3.957[9]	2.7205	2.662[9]
Cs_2PtCl_6	4.5996		3.7675		2.3561	
Cs_2PtBr_6	4.7917		3.9208		2.5096	
Cs_2PtI_6	5.0989		4.1694	4.04[10]	2.7156	2.68[10]

Table S2. Competing phases considered in calculating the chemical potential space of Rb_2PdCl_6, Rb_2PdBr_6, Cs_2PtCl_6, Cs_2PtBr_6, and Cs_2PtI_6, along with their corresponding formation energies in eV.

Limits	ΔH_f	Limits	ΔH_f
RbCl	-3.881	CsI_3	-3.612
RbBr	-3.526	CsI_4	-3.736
$PdCl_2$	-1.489	$PtCl_2$	-1.159
$PdBr_2$	-1.036	$PtCl_3$	-1.643
Rb_2PdCl_4	-9.741	$PtBr_2$	-0.686
Rb_2PdBr_4	-8.411	$PtBr_3$	-1.053
CsCl	-3.959	PtI_2	-0.647
CsBr	-3.629	PtI_3	-0.823
$CsBr_3$	-3.986	PtI_4	-1.007
CsI	-3.223	Cs_2PtCl_4	-9.706

Table S3. Calculated effective masses for Rb_2PdBr_6 and Cs_2PtI_6 compounds at the band edges using mBJ potential. The values in parentheses are calculated using PBE functional.

Compound	m_e	m_{hh}	m_{lh1}	m_{lh2}
Rb_2PdBr_6	0.84 (0.56)	2.71 (2.29)	1.56 (1.17)	1.33 (1.001)
Cs_2PtI_6	0.62 (0.46)	2.04 (1.78)	1.15 (0.91)	0.99 (0.79)

Table S4. Calculated static value of real part of dielectric function ε_0, the maximum values of ε_1 (ω), the maximum values of ε_2 (ω), the static reflectivity R (0), the maximum peak values of R(ω) the maximum peak values of optical conductivity and absorption coefficient (i.e. $\sigma(\omega)_{max}$ $\alpha(\omega)_{max}$) for A_2BX_6 (A= Rb, Cs; B= Sn, Pd, Pt; X= Cl, Br, I) compounds in the range of 0-10 eV.

	Rb_2SnBr_6	Rb_2SnI_6	Rb_2PdCl_6	Rb_2PdBr_6	Rb_2PdI_6	Cs_2PtCl_6	Cs_2PtBr_6	Cs_2PtI_6
ε_0	2.68	3.88	3.10	4.32	6.76	2.53	3.27	4.61
$\varepsilon_1(\omega)_{max}$	4.97	5.98	6.69	7.95	9.90	5.7	5.8	7.4
$\varepsilon_2(\omega)_{max}$	3.98	5.82	6.57	6.86	9.54	6.38	6.05	7.24
R(0)	0.05	0.10	0.07	0.12	0.19	0.05	0.08	0.133
R(ω)	0.23	0.28	0.64	0.51	0.35	0.54	0.48	0.32
$\sigma(\omega)_{max}$	5717	5963	5575	6266	7443	5815	5769	6985
$\alpha(\omega)_{max}$	123.3	150.2	90.03	121	152.8	94.6	132.4.8	149.8

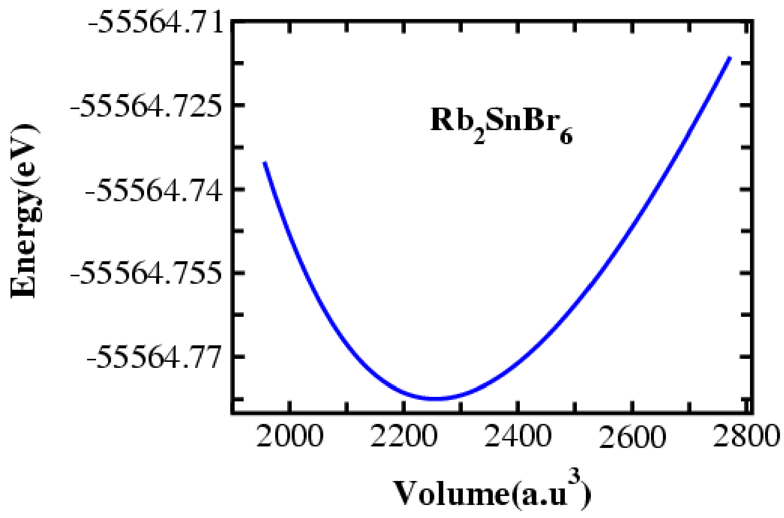

Figure S1. The total energy *vs* volume plot for representative Rb_2SnBr_6 perovskite calculated with PBE-GGA.

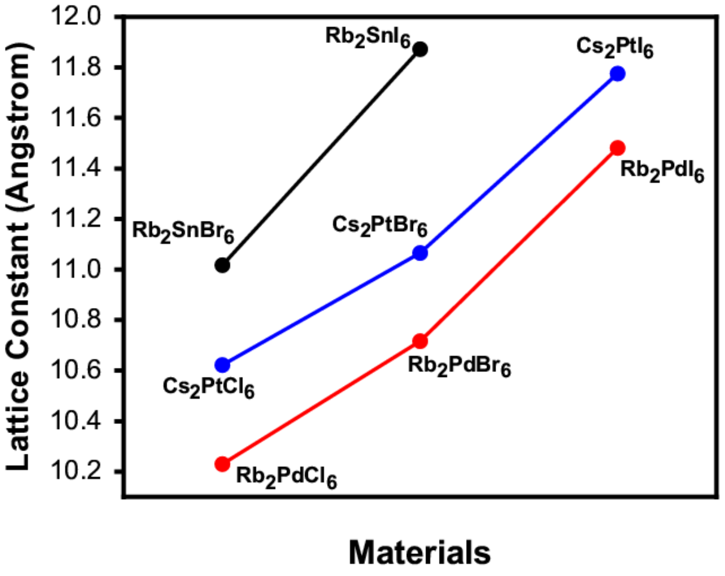

Figure S2. Periodic variation of the lattice constants with the halogen atoms.

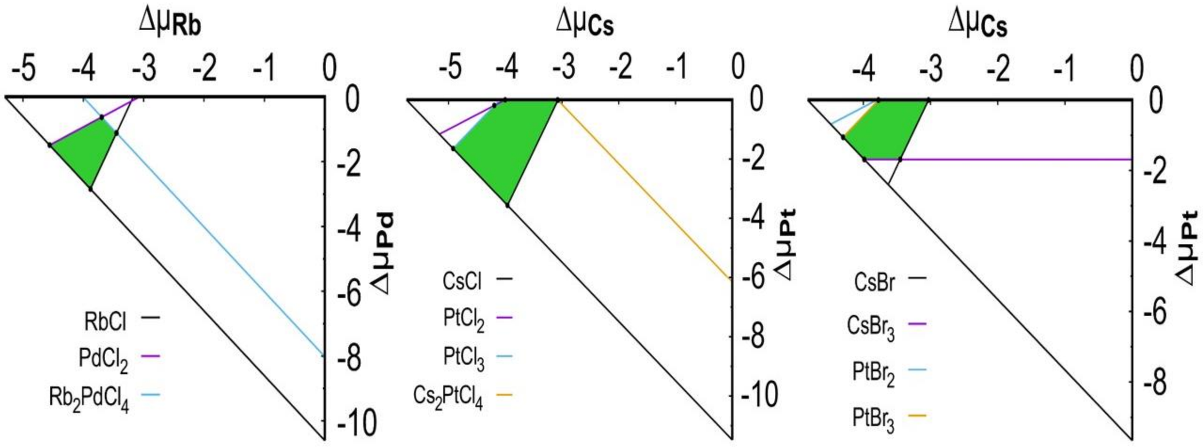

Figure S3. The stability diagrams of Rb_2PdCl_6, Cs_2PtCl_6, and Cs_2PtBr_6. Each line in the diagrams indicates a known competing phases; in each case the stable region is indicated by the green polygon.

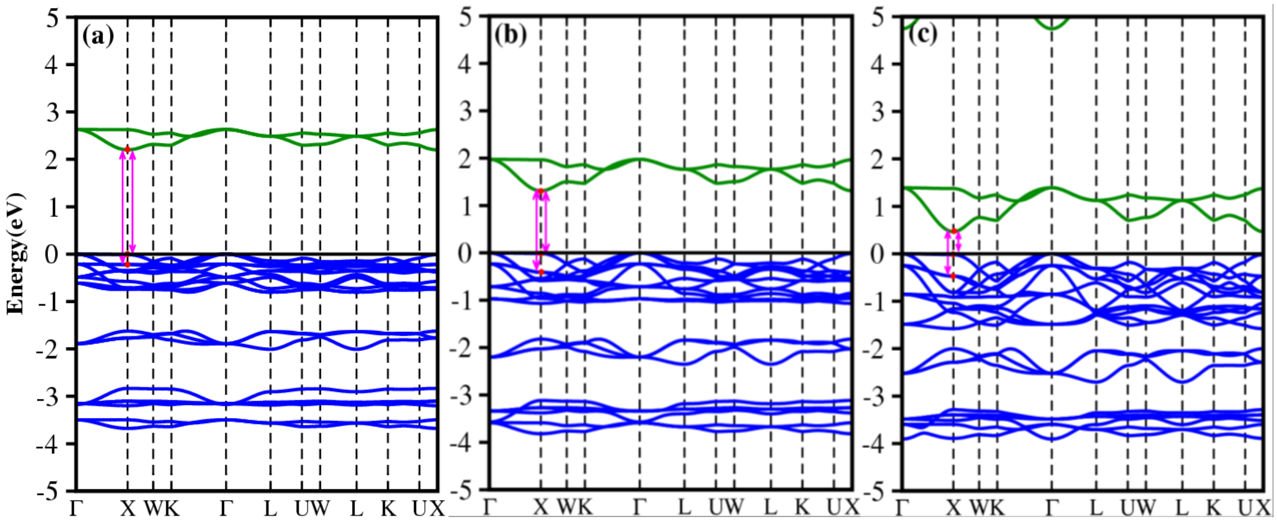

Figure S4. The calculated band structure of Rb_2PdCl_6, Rb_2PdBr_6, and Rb_2PdI_6 with mBJ potential.

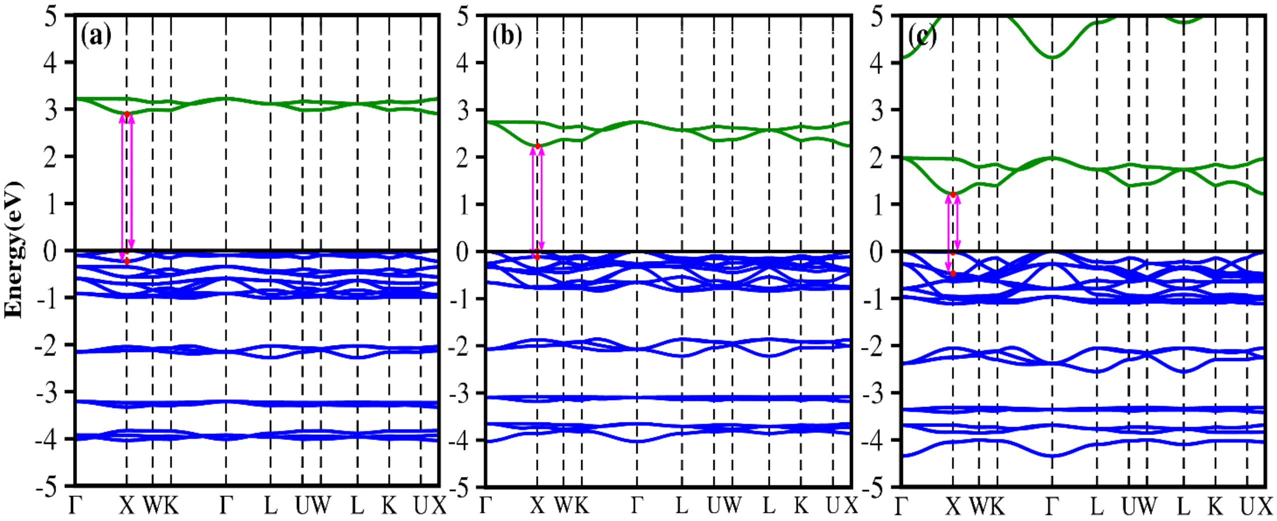

Figure S5. The calculated band structure of Cs_2PtCl_6, Cs_2PtBr_6, and Cs_2PtI_6 with mBJ potential.

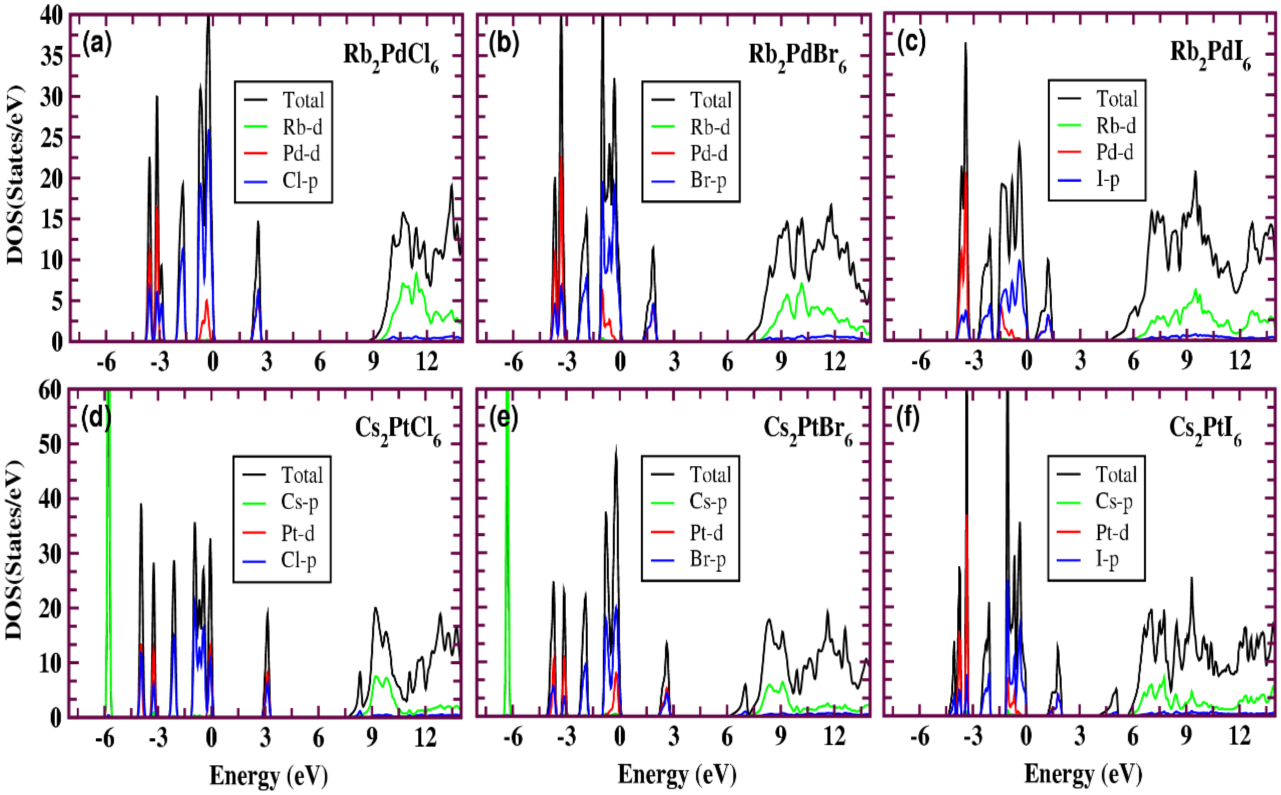

Figure S6. The total and partial density of states (DOS) for (a) Rb_2PdCl_6, (b) Rb_2PdBr_6, (c) Rb_2PdI_6, (d) Cs_2PtCl_6, (e) Cs_2PtBr_6, and (f) Cs_2PtI_6, computed using mBJ potential. The Fermi level is set to 0 eV.

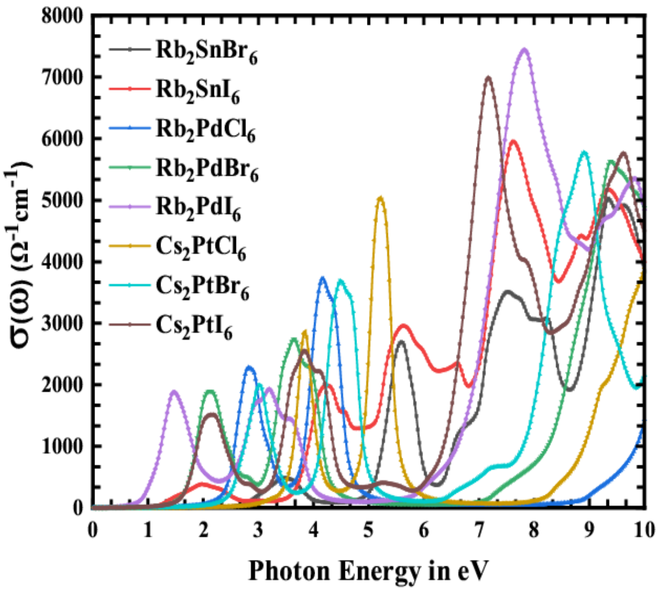

Figure S7. Optical conductivity for A_2BX_6 (A= Rb, Cs; B= Sn, Pd, Pt; X= Cl, Br, I) computed using mBJ functional.

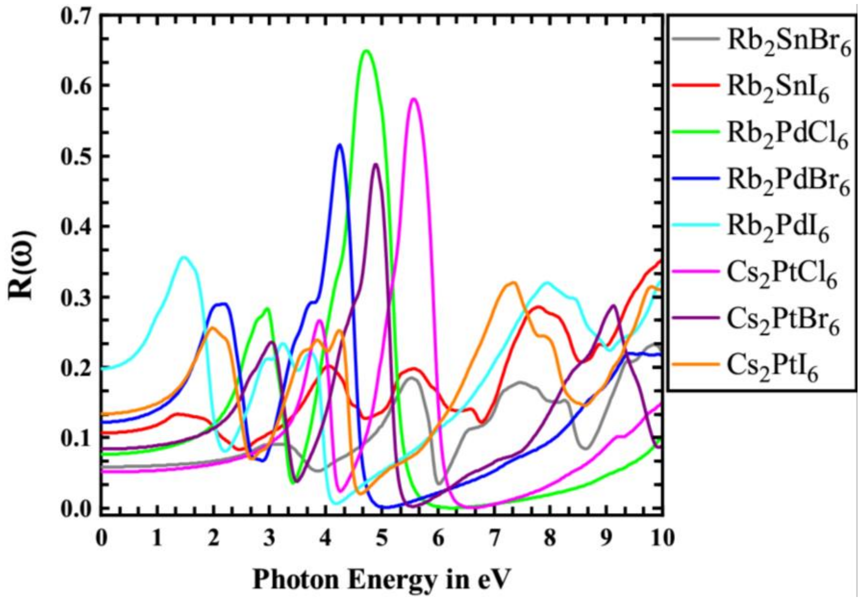

Figure S8. Reflectivity spectra *vs* incident photon energy of perovskites A_2BX_6 (A= Rb, Cs; B= Sn, Pd, Pt; X= Cl, Br, I).

References

1. Goldschmidt, V.M. Die gesetze der krystallochemie. *Naturwissenschaften* **14**, 477-485 (1926).
2. Shannon, R.D. Revised effective ionic radii and systematic studies of interatomic distances in halides and chalcogenides. *Acta Crystallogr. Sect. A* **32**, 751-767 (1976).
3. Green, M.A. Photovoltaic principles. *Physica E Low Dimens. Syst. Nanostruct.* **14**, 11-17 (2002).
4. Travis, W., Glover, E., Bronstein, H., Scanlon, D. & Palgrave, R. On the application of the tolerance factor to inorganic and hybrid halide perovskites: a revised system. *Chem. Sci.* **7**, 4548-4556 (2016).
5. Tang, L.-C., Chang, Y.-C., Huang, J.-Y., Lee, M.-H. & Chang, C.-S. First principles calculations of linear and second-order optical responses in rhombohedrally distorted perovskite ternary halides, $CsGeX_3$ (X= Cl, Br, and I). *Jpn. J. Appl. Phys.* **48**, 112402 (2009).
6. Ju, M.-G., *et al.* Toward eco-friendly and stable perovskite materials for photovoltaics. *Joule* **2**, 1231-1241 (2018).
7. Park, J.-S., *et al.* Electronic structure and optical properties of α-CH3NH3PbBr3 perovskite single crystal. *The journal of physical chemistry letters* **6**, 4304-4308 (2015).
8. Werker, W. Die Krystallstruktur des Rb_2SnJ_6 und Cs_2SnJ_6. *Recl. Trav. Chim. Pays-Bas* **58**, 257-258 (1939).
9. Schüpp, B., Heines, P. & Keller, H.L. Zwei neue Iodopalladate mit gleicher Summenformel: $Rb_2PdI_4{\cdot}I_2$–ein neuer Strukturtyp mit eingelagerten I_2-Molekülen–und Rb_2PdI_6. *Z. Anorg. Allg. Chem.* **626**, 202-207 (2000).
10. Thiele, G., Mrozek, C., Kämmerer, D. & Wittmann, K. Über Hexaiodoplatinate (IV) M_2PtI_6 (M= K, Rb, Cs, NH_4, Tl) Darstellungsverfahren, Eigenschaften und Kristallstrukturen/On Hexaiodoplatinates (IV) M_2PtI_6 (M= K, Rb, Cs, NH_4, Tl)-Preparation, Properties and Structural Data. *Z. Naturforsch. B* **38**, 905-910 (1983).
